# Multidisciplinary team meetings and their impact on workflow in radiology and pathology departments

**DOI:** 10.1186/1741-7015-5-15

**Published:** 2007-06-13

**Authors:** Bridget Kane, Saturnino Luz, D Sean O'Briain, Ronan McDermott

**Affiliations:** 1 Department of Computer Science, Trinity College, Dublin, Ireland; 2 Trinity Centre for Health Sciences, St. James's Hospital, Dublin, Ireland; 3 Central Pathology Laboratory, St. James's Hospital1, Dublin, Ireland; 4 Diagnostic Imaging Department, St. James's Hospital, Dublin, Ireland

## Abstract

**Background:**

The development of multidisciplinary team meetings (MDTMs) for radiology and pathology is a burgeoning area that increasingly impacts on work processes in both of these departments. The aim of this study was to examine work processes and quantify the time demands on radiologists and pathologists associated with MDTM practices at a large teaching hospital. The observations reported in this paper reflect a general trend affecting hospitals and our conclusions will have relevance for others implementing clinical practice guidelines.

**Methods:**

For one month, all work related to clinical meetings between pathology and radiology with clinical staff was documented and later analysed.

**Results:**

The number of meetings to which pathology and radiology contribute at a large university teaching hospital, ranges from two to eight per day, excluding grand rounds, and amounts to approximately 50 meetings per month for each department. For one month, over 300 h were spent by pathologists and radiologists on 81 meetings, where almost 1000 patients were discussed. For each meeting hour, there were, on average, 2.4 pathology hours and 2 radiology hours spent in preparation. Two to three meetings per week are conducted over a teleconferencing link. Average meeting time is 1 h. Preparation time per meeting ranges from 0.3 to 6 h for pathology, and 0.5 to 4 for radiology. The review process in preparation for meetings improves internal quality standards. Materials produced externally (for example imaging) can amount to almost 50% of the material to be reviewed on a single patient. The number of meetings per month has increased by 50% over the past two years. Further increase is expected in both the numbers and duration of meetings when scheduling issues are resolved. A changing trend in the management of referred patients with the development of MDTMs and the introduction of teleconferencing was noted.

**Conclusion:**

Difficulties are being experienced by pathology and radiology departments participating fully in several multidisciplinary teams. Time spent at meetings, and in preparation for MDTMs is significant. Issues of timing and the coordination of materials to be reviewed are sometimes irreconcilable. The exchange of patient materials with outside institutions is a cause for concern when full data are not made available in a timely fashion. The process of preparation for meetings is having a positive influence on quality, but more resources are needed in pathology and radiology to realise the full benefits of multidisciplinary team working.

## Background

In recent years there has been significant growth in multidisciplinary team working [1] as a result of increasing specialisation, advances in medical technologies [2] including teleconferencing, and recommendations by respected agencies [3-7]. Multidisciplinary teams and their meetings now occupy a central role in developed health systems [8]. Large teaching hospitals, in particular, are witnessing these developments and the increase in the number of multidisciplinary team meetings (MDTMs) that results from them.

Pathologists and radiologists are important contributors to multidisciplinary teams [9], and the role of these two specialists is different from other multidisciplinary team participants in that they often belong to several groups and actively contribute in many MDTMs. Radiology and pathology, with respect to their work organisation and input to meetings, have more similarities than differences. This paper analyses the work associated with MDTMs (and clinical pathology or radiology meetings) and identifies emerging effects in relation to time management, scheduling and pre-meeting work that the development of such meetings have on radiology and pathology departments. We show that senior staff in radiology and pathology now spend almost 20% of their time either preparing for, or participating in, meetings with clinical staff.

Some of the demands for more MDTMs are attributed to developments in teleconferencing technology that have facilitated changes in service structures and the extension of multidisciplinary teams geographically [10,11]. Consultations are taking place through MDTMs, patient care pathways are being tailored and treatment coordinated through case discussions. A change in patient referral patterns is noted here resulting from the availability of the meetings and the interactions via teleconferencing, and we expect this trend to continue, with consequences for current work practices.

While this study is confined to one hospital – a 963 bed facility where over 2000 new cancers are treated every year – these results will have resonance for others, particularly tertiary referral centres and teaching hospitals, who are likely experiencing similar changes. Although the demand for meetings has been growing over recent years, the issues surrounding MDTMs for service departments such as radiology and pathology have not been formally articulated nor quantified until now. The demand for meetings continues to grow, given the success of current practices. Requests are ongoing for new MDTMs, more lengthy MDTMs and more in-depth discussion on a larger number of patients. But for the full benefits of MDTMs to be achieved, improved solutions are needed to overcome the difficulties being experienced that are documented in this study.

The MDTM can be described as a system, or coordination mechanism, across functional departments at the hospital that adds dependability to the overall patient management and diagnostic process (discussed in [12]). This study of the work involved in preparation for MDTMs is part of a larger ongoing study on multidisciplinary medical team working. Quantitative investigation of the impact of MDTMs on quality is underway.

### Local practice

Terminology for meetings between radiology, pathology and clinical teams varies. The term multidisciplinary is applied to team meetings where both pathology and radiology contribute and at which physicians, surgeons, radiation and clinical oncologists, at a minimum, have input. Clinical-pathology and clinical-radiology conferences (CPCs and CRCs) are held between clinical teams and pathology and radiology staff respectively. Internal departmental processes are the same for MDTMs and CPCs or CRCs.

As part of good practice policy, radiology images [7], biological material and all reports are reviewed prior to discussion at MDTMs. This review is important in quality management. Meetings serve an educational role as well as having organizational and patient management functions.

For all meetings, a list of patients to be discussed is circulated in advance to team members. Patient samples and images are located if not on the Picture Archiving and Communications System (PACS), along with reports, for review by radiologists and pathologists, prior to the meeting. Any patients who have had radiological imaging or tissue sampling performed elsewhere will have those items reviewed in conjunction with any current materials.

## Methods

Participant observation of work practices, semi-structured interviews, literature review and the analysis of organizational records for quantitative data provided the material for this study. Meeting agendas and notes, radiological images and pathology samples used at meetings were examined. Internal pathology department records for 2003 enabled comparison with November 2005 data for pathology.

Over 240 h of meetings were observed over 22 months. Semi-structured interviews were conducted with consultant and non-consultant medical staff, nurses, technical and support staff.

Meeting preparation work by medical staff was self-reported. Senior radiology and pathology staff were asked to prospectively note the time they spent on meeting preparation for the month of November 2005. At the end of that month, the time spent was reported in interview.

Figures quoted here are agreed averages and take account of the mixture of cases one would expect to encounter (biopsies, resections, type of image sets and repeat review) for an average meeting. Technical and administration work estimations are not fully quantified here. This paper focuses on the time spent by senior medical staff in radiology and pathology, i.e. at specialist registrar and consultant level.

Special focus was given to the month of November 2005, a 30-day month with 22 working days (Monday to Friday inclusive). The numbers and types of meetings held, the patients discussed, the radiological images used and pathology samples reviewed were counted. November 2005, was a typical working month and hence gives a representative view of MDTMs at St. James's hospital. Grand rounds and internal meetings, as part of postgraduate specialist training, were excluded.

The patient cases discussed at the selection of meetings involving radiology and pathology (Table 1) were also examined to measure the frequency of cases being discussed within the same type of MDTM and across different MDTMs for the period under study. For a sample of the MDTMs in November 2005, a more detailed examination was conducted to quantify the pathology specimens and radiological images reviewed that were the product of procedures performed elsewhere. Patient referral patterns were noted.

**Table 1:** Meeting schedule overview including all MDTMs, CPCs and CRCs

**Period**	**Description**	**Time** ** (mean h, Nov)**	**Duration** ** (mean h, Nov)**	**Preparation (h)**		**Mean no. cases**
				**Pathology**	**Radiology**	

Mon	Respiratory	800	2 × 4	6	2	22.25
Mon	Gynaecology	800	1 × 4	5	2	9
Mon	Hepatology CRC	1 300	1 × 4	--	0	Cancelled
Tues	Breast	800	1 × 5	3	1	14
Tues	Dermatology CPC	1 245	1 × 4	2.5	--	7
Tues	Haematopathology CPC	1 400	1 × 5	2	--	9.6
Wed	Haematology CRC	800	0.5 × 5	--	2	8
Wed	GI1 CRC	830	0.75 × 5	--	3	15
Wed	Hepatology CPC	1 300	1 × 5	0.33	--	6.2
Wed	CMD2 CPC	1 400	1 × 5	1	--	27.4
Wed	Oncology CPC	1 600	1 × 5	1	--	3.5
Thurs	HNT3 CRC	715	0.75 × 2	--	1	8
Thurs	HNT CPC	745	1 × 2	3.5	--	15
Thurs	GI oncology	730	0.75 × 3	3.5	2	6.7
Thurs	Lymphoma	815	0.75 × 4	2	1	5.5
Thurs	Gerontology CRC	830	0.75 × 4	--	1	10
Thurs	Rheumatology CRC	915	0.75 × 4	--	0.5	10
Thurs	Medical GI CPC	1 315	1 × 4	2	--	8
Thurs	Med Oncology CRC	1 330	0.75 × 4	--	4	15
Fri	Urology	915	0.75 × 2	2.5	1	4.5
Fri	Neurology CRC	1 300	1 × 4	--	0	Cancelled
Twice weekly	Skin cancer CPC	1 415	1	2.5	--	47
Monthly	Oral med./surgery CPC	1 300	1	3.5	--	10
Monthly	Renal pathology CPC	800	1	0	--	Cancelled
Monthly	Infectious Disease CRC	1 400	1	--	1	9
Monthly	Death Conference	800	1	3.5	1	2
Quarterly	Endocrinology CRC	1 230	1	-	0	Not due in Nov.
Occasional	Maxillo-Facial CPC	1 400	1	1.5	--	7

	Total h, November 2005		83.5	140.15	84	914.2

Approval for this study was given by the St James's Hospital and Adelaide and Meath Hospital (incorporating the National Children's Hospital) Joint Research Ethics Committee.

## Results

Table 1 gives an overview of the meeting schedule, the preparation involved and the mean numbers of patients discussed. The table includes all MDTMs, CPCs and CRCs. There are six meetings scheduled per week that involve both radiology and pathology together (MDTMs). There are an additional eight CRCs and seven CPCs per week. There are also twice monthly, monthly and other less frequent meetings. Table 1 summarises the meeting schedule, and those meetings held in November 2005. A total of 94 meetings were scheduled and 81 held that took 75.5 h. Eight CRCs, two CPCs and two MDTMs were cancelled due to unavailability of key personnel and one MDTM was cancelled because late circulation of the agenda did not allow adequate time for meeting preparation. Pathology was represented at 55 meetings that lasted a total of 57.75 h, while radiology was represented at 52 meetings that took 42.5 h in total for the month under study. Table 2 summarises the time spent in preparation and at meetings during the month of November 2005. Reported values take account of situations where images or samples might be quickly reviewed, might not be considered relevant to the discussion tabled, and hence would not be presented to the meeting.

**Table 2:** Time spent at, and in preparation for, meetings during one month

**Department**	**Meeting time** ** per month (h)**	**Average preparation time** ** per h meeting**	**Total h** ** per month**
Radiology	42.5	2	126.5
Pathology	57.75	2.4	197.9

At least one consultant radiologist and pathologist always attends an MDTM, and often two are designated members of a single multidisciplinary team (and hence two regularly attend). The total compliment of consultant staff in pathology and radiology is 7.9 and 9 full time equivalents (FTE) respectively, and almost 0.5 FTE is spent in attendance at meetings for each department.

Meetings held with either radiology or pathology, (CRCs and CPCs), represent situations where either (a) radiology or pathology serve more important clinical needs, (b) there is a high volume of work with limited discussion time, or (c) there is no time within the schedules for the people involved to be in the same place at the same time. Examples are: in vascular surgery, imaging is of key importance and pathology is not so significant; dermatologists rely heavily on pathology but do not have a great need for radiology. Dermatology hold two meetings: a weekly meeting to review non-cancer pathology and a second, twice monthly, to deal with skin cancer. For head, neck and thyroid (HNT), it was not possible to find a time for everyone to meet together, so the HNT specialists met with radiology and pathology on alternate weeks (which was less than satisfactory).

### The pre-meeting review

All images and tissue samples are reviewed prior to discussion at meetings, separately and independently of the main work process, regardless of whether the materials were produced internally or externally to the hospital. This review applies for all meetings; it is ancillary and complimentary to the original work of making the primary diagnosis. In the review, the primary diagnosis is confirmed and refined if necessary. The review process satisfies a training and education function for pathologists and radiologists, as well as an important quality assurance role within the department [7].

The review of external work serves as a check on the original report, both for opinion differences and expression. For radiology, the full image set is rarely available. For pathology, slides and processed tissue are received from referring institutions.

The internal review serves an internal quality assurance function within pathology and radiology. The material to be discussed is reviewed by the consultant, often in association with a registrar, and material for presentation is selected and prepared. Typically, the person reviewing the specimen for discussion is not the same person who undertook the initial examination within the main work process. Discrepancies in reports will be discussed within the department in the first instance and a revised or amended report can be issued in the light of those discussions. Practice differs in radiology and pathology with regard to the issue of contradictory reports, particularly in the absence of full image sets, and a formal policy remains to be agreed and established.

The issues of internal quality assurance for radiology and pathology will not be further covered here. It is sufficient to note that the practice of a second review of materials is a recognised method of improving quality in work processes [13]. In November 2005, the pathology department reviewed tissue samples on 628 patient cases. This represents almost 47% of the total caseload for that month. While an exact figure is not available for radiology for November 2005, the radiology department performs approximately 10 CT thorax scans per week, and reviews approximately 25 CT thorax scans for a Monday morning respiratory meeting. Approximately 2200 imaging studies are performed each week, and it is therefore conservatively estimated that between 10 to 15% of the radiology workload is reviewed in preparation for MDTMs.

### Workload

The workload for senior medical staff associated with attendance, preparation and review of materials pre-MDTMs is given in Table 1. In addition to the medical staff workload, administrative and technical staff are also involved in preparation for meetings. Four clerical staff were appointed in March 2005 to coordinate the pre-meeting work associated with MDTMs, (circulate agendas, locate radiological images and pathology slides) and to take notes at the meetings. The MDTM coordinators also liaise with outside agencies to exchange images and pathology material for review at the meetings. Administrative staff in radiology and pathology are also involved in meeting preparation and in the receipt and packaging of review material for postal services.

Approximately 4 min of pathology clerical time is used when an in-house patient case is identified for discussion at a forthcoming MDTM. When outside material is sent for review, an additional 10 min is spent opening the package and logging receipt, and packaging for subsequent return by post. Additional technical time is not always associated with MDTMs, but when external tissue is sent for review additional tissue sections are often required and extra special stains can be required, mostly relating to immunohistochemical methods. In November 2005, 55 external pathology cases were reviewed for MDTMs, of which 21 had special techniques applied. Overall, approximately 15 min administrative work in pathology is associated with each internal patient to be discussed at a meeting. This time will be longer if histological slides have to be retrieved from old archives. In radiology, considerable time can be spent searching for images if the image is not available on the recently installed PACS, or if images need to be retrieved from outside institutions.

### Meeting times and places

**Figure 1:**
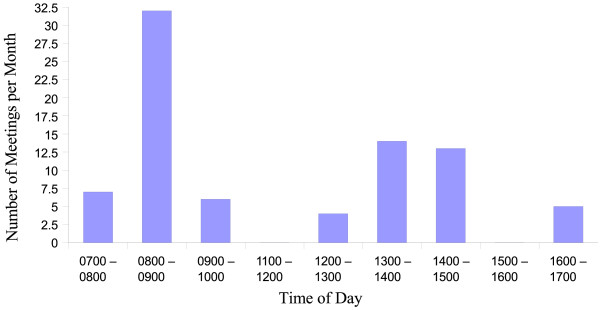
Timing of multidisciplinary meetings. The figure shows that, with five exceptions (out of a total of 28), all of the meetings are held in the early morning or at lunchtime.

Similar to findings from other reviews [14,15], with five exceptions (from a total of 28), all of the meetings are held in the early morning or at lunchtime. Figure 1 shows the timing of the MDTMs in November 2005. For most MDTMs, the scheduled meeting duration is 1 h, but meetings frequently take longer. Because of the high demand for meetings to be held between 7.30 and 9.00 am, some groups have agreed to curtail their discussion to accommodate another group. With two exceptions, meetings are held on-site. Three meeting rooms are used, one of which is equipped with the Telesynergy^® ^teleconferencing workstation (supplied by the Centre for Information Technology, National Institutes for Health, Bethesda, USA). Out of the six MDTMs held each week, with both radiology and pathology present together, there are three videoconferencing links to remote hospitals using the Telesynergy^® ^workstation. The GI group link weekly with one other centre. The respiratory MDTMs link to two distant hospitals concurrently, twice monthly, while the lymphoma MDTMs link with two hospitals, one at a time over successive weeks, on a once-monthly basis for each remote hospital.

The coordination of individual schedules, outpatient clinics and theatre sessions for all of the individuals involved in MDTMs requires high levels of cooperation, and sometimes it is not logistically possible to reconcile all the schedules involved. Several specialist appointments are contracted over multiple hospitals and, for a meeting to be arranged, schedules must be coordinated over the several hospitals and many teams affected.

Teleconferencing to some distant hospitals was initiated over 2004 and 2005. In November 2005, there were 10 h of MDTMs conducted with a teleconference link involving three other hospitals. It is anticipated that the amount of meetings held over teleconferencing links will increase in future, both in the frequency and duration for existing associations, and as teamworking develops over multiple sites.

### The growth in demand for meetings

The number and frequency of MDTMs is increasing. Between 2003 and 2005, four new MDTMs were initiated and others increased in frequency. Since 2003, there has been an increase of 50% in the amount of time spent at meetings (from 9 to 13.5 h per week) and a resultant 47% increase in the number of patient cases discussed (from 426 to 626). More meetings are in the planning stages. The advent of teleconferencing has also developed the MDT services over wider geographical areas [10], and meetings held via teleconference have increased from 0 h in 2003 to 2 h, of the 13.5 h, per week in November 2005.

It is expected that this trend for increased frequency and duration of meetings will continue, more meetings will be held via teleconference, and more patients will be managed through team meetings in the future.

### Patients, images and tissues

Analysis of a subset of meetings is summarised in Table 3. This representative sample of MDTMs held in November 2005 excludes meetings held via teleconference and accounts for 40% of the MDTMs held that month. The aim was to quantify details on the items discussed at the MDTMs, i.e. the number of patient cases who had radiological imaging or pathology samples for discussion; the number of radiology or pathology items for review and the number of those items that were the result of procedures performed at outside institutions.

**Table 3:** Analysis of the ratio of specimens per patient and the proportion of externally-produced material

**Proportion of Patient cases** ** with radiology/pathology**		**Ratio of** **patients: specimens/images** **for examination**	**Proportion of ** **externally-produced material**
Radiology	0.75	1.62	47%
Pathology	0.81	2.39	19%

The number of patients discussed and the number of radiology images and pathology samples can vary widely between meetings. Table 3 gives the average ratio of image studies per patient. The number of image studies and tissue samples per patient will depend on the disease, its duration and complexity. In radiology, for example, respiratory patients typically have chest radiographs, CT and PET scans, while most patients with breast lesions have mammograms and ultrasounds only. In agreement with other reports [16,17] it was found that radiology films are regularly unavailable for discussion at meetings. Some hospitals report as much as 20% of images are missing when required[16]. For pathology, a distinction has not been made between pathology biopsies and large resection specimens, but meetings with large resection specimens for discussion involve more pathology preparation. Digital pictures are taken at gross dissection, as well as photomicrographs of stained sections, as part of the pathology pre-meeting preparation.

Table 3 also shows how almost 50% of all the images and approximately 20% of all pathology samples reviewed were from outside institutions. The figure for radiology images would have been much higher than 47% if all images had been available for discussion. The cases represented in Table 3 were current patients of the hospital who underwent some investigative procedures elsewhere prior to referral. When conducting this review summarised in Table 3, instances were noted of patient imaging and pathology being reviewed in advance of the patient being examined by the clinical specialists at the hospital (i.e. on receipt of the referral letter). These discussions assisted in the prioritisation of a patient procedure or appointment at an outpatient clinic.

For patients from other hospitals being discussed via teleconference, all the imaging and pathology sampling is performed by others and is reviewed at St. James's prior to the teleconference discussion. Discussion on remote hospital cases via teleconference sometimes resulted in patients not being transferred to the hospital for further assessment (which would have happened if there had not been a meeting). Teleconference case discussions also directed the patient towards the most appropriate clinician for review or treatment. A patient can be discussed at more than one type of meeting, or at a number of meetings. Patients can also be discussed at more than one meeting of the same type. It is normal practice for patients to be 'staged' and managed through MDTMs. If the patient is discussed on initial diagnosis and it is decided to proceed to surgery, the patient will be presented again to the meeting following the procedure, to review the pathology and imaging (if appropriate) and decide on the next step in management.

The patients discussed at MDTMs involving both radiology and pathology were also examined to determine how many patients featured in multiple discussions. There were three gynaecology and eight respiratory patients discussed twice at their respective MDTMs. A single patient was referred from the GI MDTM to the respiratory MDTM being held 4 days later, and thus was discussed by two different teams (GI and respiratory). For lymphoma, urology and gastro-intestinal (GI) MDTMs, no patient was discussed on more than one occasion. For breast patients it is normal practice to discuss 'clinic' cases (from the lump assessment clinic) and 'histology cases' at breast MDTMs. Histology cases are those referred for surgery from the assessment clinic service that have been scheduled for review following their resection. There were 68 case discussions in November representing 53 women, 15 of whom were reassessed following lump resection. In the respiratory meetings, there were 87 patient case discussions in November 2005 on 79 patients. Eight patients were discussed at two meetings and one patient was discussed three times. There was one example of a patient being placed on the agenda but the discussion was deferred until the following week. Of the 79 respiratory patients, 55 were 'new' in-house cases and a further 10 were from outside institutions. Thus, approximately 10% of case discussions for respiratory and breast are repeat review discussions within the same month. Review time is not the same for all cases, depending on specimen type (biopsy or complex resection) or the number and type of image sets to be examined. Times given for MDTM preparation takes these variations into account.

## Discussion

The continuing demand for the review of pathology and radiology findings at meetings is a testament to the perceived value and success of such reviews. One of the experienced benefits of MDTMs is the opportunity for clinicians, radiologists and pathologists to meet together and build common ground with respect to terminology and expression in formal reports. Communication has improved between radiologists, pathologists and clinicians, both in the provision of pertinent information to radiology and pathology at the time of request and the provision of formal reports from radiology and pathology whose meaning is clear to clinicians. Furthermore, technological developments in radiology and pathology makes the choice of investigation and the interpretation of results more complex than in the past, so the meetings serve as an important opportunity for updating professional knowledge and continuing professional development. Table 4 summarises the benefits and challenges identified with our meeting practices.

**Table 4:** Summary of key benefits and challenges associated with MDTMs

**Benefits**	**Challenges**
Clinical, Pathology, Radiology correlation	Scheduling
Refinement of pathology or radiology report	Timing
Definitive diagnosis, disease stage established	Duration
Improved decision making	Resources
Coordination of patient management	Contractual arrangements
Interprofessional communication	Co-ordinating materials
Feedback and peer review	Pre-meeting review
Local policy development	Reviewing partial images from outside institutions
Preparation improves Radiology and Pathology QA	Formal reporting on reviewed material
Data collection for audit	
Education	

Four important issues emerge in fulfilling the demand being placed on pathology and radiology departments: (1) time spent at meetings, (2) timing and coordination of meetings, (3) time spent in preparation for meetings, including review and the retrieval of slides, reports and images, and (4) review of externally-produced images and tissue material.

### Time and resources

The work associated with being part of a multidisciplinary team is a significant process that has emerged within pathology and radiology departments and has not been clearly identified up to now. There is an accepted belief that MDTMs are an improvement in the patient diagnosis and management process and integral to quality systems within the hospital. However, the practice has grown almost surreptitiously, outside of the normal working day in many cases. In other words, development of MDT working advanced more rapidly than the rate at which resources were designated. In November 2005, the 83.5 h of meeting time alone (Table 1) represents almost three FTE, or 20% of allocated resources in radiology and pathology. Of the 29 scheduled meetings, 21 are held outside of the contractually obligated working hours of staff, and much of the meeting preparation is also conducted outside of the 'normal' working day. This time is not readily reimbursed, and while staff express high satisfaction levels because of interactions at meetings, additional strain is placed on the resources for routine work.

The increased workload has placed considerable pressure on the radiology and pathology departments and this is currently being absorbed by an increase in working hours by many staff. Current practice is reliant on goodwill and professional duty of care and is not a sustainable solution to the ever-increasing workload of MDTMs in the long term.

The demand for meetings has increased since 2003 and is likely to continue to increase, given the recommendations of professional and regulatory organisations to include multidisciplinary team working in patient practice management protocols. At this time, at least two additional meetings are in the planning stage and more have been requested (such as a monthly TB meeting). The number of meetings reported here would be greater if requests by clinical staff had been facilitated, and seem more than the numbers of meetings reported by others [18]. Currently, several groups curtail their MDTM time because of time pressures in schedules. Several groups have requested that more time be made available and there is a need felt among many that more patients should be discussed in more depth. However, time constraints have dictated that discussion is highly structured and the number of cases is maintained at current levels.

The practice of producing digital image presentations of pathology material is growing in popularity. While it adds time to preparation for pathology, valuable time is saved at the actual meeting (i.e. loading of the correct slide, locating and focusing on the feature to discuss).

### MDTMs, times and places

Each multidisciplinary team wishes to schedule meetings with radiology and pathology to suit their routines. For radiology and pathology it is a problem to satisfy the requirements of multiple teams within a narrow timeframe. As shown in Figure 1, teams find that the only time that they can meet together is early in the morning or at lunchtime, outside of the 'normal' routine, because individuals within the team have clinics or theatres scheduled during the 9-to-5 periods and the only time when all members are free is outside of their 9-to-5 routines.

As well as the difficulties of coordination and cooperation within the hospital challenges are experienced in managing multidisciplinary team working across several hospitals. Some specialists serve more than one hospital. For example, the cardiothoracic surgeons are contracted to two hospitals and are members of a multidisciplinary team at each hospital; consequently, the schedule in one hospital impacts on the other. When one change is made, for example in an outpatient clinic, there will be significant impact on many schedules in different hospitals. Sometimes it is not possible to build schedules to suit the many people and systems affected.

The development of directorate structures has helped group some services, and helped in scheduling related activities. But pathology and radiology contribute to every team and need to find better ways to work within clinical teams. With developments in imaging modalities and molecular techniques and the capabilities for image-guided tissue sampling, pathology and radiology are co-ordinating more in service development and now occupy a more central role in clinical service delivery.

A further development since 2003 is the advance in teleconferencing technology that has facilitated the extension of multidisciplinary team working to distant hospitals where the full range of expertise is not available. Co-ordinating schedules for MDTMs via teleconference to synchronise with external institution schedules is an additional challenge. Furthermore, the coordination of sending, receiving and reviewing radiology and pathology before teleconference discussions is proving more difficult than anticipated and requires dedicated resources. While teleconferencing technology has facilitated development of some multidisciplinary team services, it has not resolved the problem of synchronous communication between multiple groups with different agendas [12]. Indeed, technology, such as teleconferencing, might have increased expectations as to the ease with which specialist services can potentially be delivered and imposed additional pressures on staff at specialist (and general) centres that were not anticipated.

## MDTM preparation

While the preparation time overlaps with the internal quality assurance process, it is proving difficult to accommodate both the main workflows and the meeting preparation workflow in parallel in the normal working day. Similar to other reported experiences [19], discrepancies in reporting of material from outside sources justifies the review of all externally-produced images and tissue samples. Although a large proportion of the review work is conducted in the early morning and late evening, routine processes are also impacted. As well as registrars-in-training and consultant staff being affected, the administrative work associated with meetings is usually underestimated.

There can be multiple pathologies in a patient, and for some patients situations change and they need to be discussed again. Our figures suggest that overall over 30% of patients reappear at any one conference, (e.g. sequential breast) and <1% are discussed at different conferences (e.g. respiratory and GI). Repeat reviews frequently focus on a limited aspect of the case, possibly omitting either radiology or pathology, and thus can take a fraction of the time of a first review (figures quoted in Table 1 take repeat reviews into account).

### External production of images and tissue material

The issue with the review of external images and tissue material has three aspects. Firstly, our data are highlighting changing trends in patient diagnostic pathways. It is proving a challenge to develop systems that coordinate the exchange and review of materials within the current schedules. Some patients have imaging performed elsewhere while waiting for appointments, and some patients can be managed without need to attend the hospital as a consequence of discussion at a meeting. Both the existence of the meeting and teleconferencing are influencing these changing work practices. On occasions, patient cases are discussed prior to their attendance, following review of radiology and pathology performed elsewhere. If the review and discussion is satisfactory, the patient might not need to attend at all. This change in patient referral practices has implications for traditional models of resource allocation that are based on patients' hospital attendances and without account of consultation and advice services given on materials generated elsewhere.

Secondly, the lack of standardisation of image production and tissue sections can result in less than optimal results (interpretations) within radiology and pathology. The exchange of radiology image sets is less satisfactory than having full scan data available for review. As well as the loss of information, local protocols and equipment settings might produce images that differ, and lack of inter-laboratory standardisation of laboratory staining can cause some discrepancies in interpretation and pathology reporting. Despite the limitations, there is an ultimate benefit to the patient because of the discussion at the MDTM and often those materials are all that are available.

The third issue is the logistics of exchange and sharing of externally-produced material. The review of patient images is greatly facilitated with the introduction of a PACS. However, there remain significant logistical problems associated with the exchange of patient images from outside institutions in the absence of a suitable infrastructure. Exchange and sharing of tissue and cell samples are likely to remain an issue.

### Recommendations

For MDTMs to be fully integrated into the 'normal' working of hospitals, scheduling issues need to be resolved and meetings need to be recognised as part of the working day. It would be desirable for meetings to be allocated 'protected time' and considered an integral part of a clinicians workload, as practiced in some jurisdictions [14]. It is generally understood that coordination mechanisms in organisations add cost over purely functional structures [20], and that complexity, uncertainty and interdependence of work create additional information-processing demands when coordinating activities [21]. MDTMs serve to coordinate the interdependent work involved in patient diagnosis and management and can be expected to incur an additional resource cost to realise their potential benefits. Additional radiologist and pathologist staff are required to fully service the needs of MDTMs at current levels. As well as radiologist and pathologist support, additional support is needed for coordination of materials and follow-up on administrative tasks assigned at meetings. Four coordinators currently administer the seven weekly meetings listed in Table 1 that involve both pathology and radiology. Ideally, the role would be developed and there would be a designated coordinator for each meeting.

Many of the problems currently being faced in implementing MDTMs as part of good practice would be alleviated through the provision of additional resources and formalising of multidisciplinary team working into contractual arrangements.

However, resources alone will not remedy issues of timing and coordination, nor problems associated with different imaging protocols and incompatible software systems. High levels of cooperation will be needed between departments and hospitals to facilitate work schedules. Scheduling of theatre sessions, for example, will impact on pathology workflow and affect meeting schedules. Enough time must have lapsed between surgical removal of a specimen and the meeting to allow time for tissue processing and review. The lack of imaging protocols and compatible standards to support portability is an issue that might be best addressed by the development of agreed standards through representative and professional bodies.

## Conclusion

Interactions between pathology, radiology and clinical specialists at meetings add quality to the diagnosis, disease staging and patient management decisions. While the overall cost to the health service might not be significant, as the system becomes more efficient there is an additional cost that tends to be overlooked for pathology and radiology departments. MDTMs are coordinating mechanisms in the patient diagnostic and management processes and require additional resources (over purely functional structures) to operate effectively. The work involved in attending meetings and in preparation for meetings is becoming a more significant part of the work in radiology and pathology. Further demand for MDTMs is predicted, given the emphasis on multidisciplinary team working, technological developments in imaging, advances in pathology and in technologies such as teleconferencing. In order to reap the true benefits of multidisciplinary teamworking and MDTM developments, the issues highlighted here for radiology and pathology will need to be resolved.

## Competing interests

The author(s) declare that they have no competing interests.

## Authors' contributions

The study was conceived by BK and SL following an initial study of multidisciplinary team meetings. Through collaboration between all the authors, the ideas were developed and the study designed. Data were collected by BK with contributions from DSO'B and RMcD. RMcD and DSO'B contributed their expertise in the areas of radiology and pathology, respectively. DSO'B and RMcD critically evaluated draft manuscripts. SL directly supervised the work through all its stages, including contributing to manuscript revisions and the final version. BK, SL and DSO'B were involved in obtaining ethical approval. All authors approved the final version of the manuscript.

## Pre-publication history

The pre-publication history for this paper can be accessed here:


